# New Vistas for *Withania somnifera* in Signal Transduction of Inflammation and Aging

**DOI:** 10.1007/s11095-026-04060-0

**Published:** 2026-04-02

**Authors:** Eshita Sharma, Dilip Mehta, Prem Muthuraj, Sangita Panda, Saiprasad Ajgaonkar, Dishant Maniar, Aswathi Biju, Sujit Nair

**Affiliations:** 1 Phytoveda Pvt. Ltd, Mumbai, 400022 India; 2 Viridis Biopharma Pvt. Ltd, Mumbai, 400022 India; 3 Drexel University, Philadelphia, PA 19104 USA; 4 Saveetha Medical College and Hospital, SIMATS, Chennai, 602105 India

**Keywords:** age-related diseases, epigenetics, inflammaging, signal transduction, *Withania somnifera*

## Abstract

Aging in humans is a multidimensional complexity featured by systematic chronic inflammation and further accompanied by organ dysfunction, gut dysbiosis, immune senescence, and age-related diseases. Chronic inflammation in cells relates to secretory factors like the senescence-associated secretory phenotype which induces senescence in normal cells. Simultaneously, immune senescence is promoted by chronic inflammation, resulting in an impaired immune system unable to clear out senescent cells and inflammatory factors. Long term accumulation of elevated inflammatory factors in cells causes organ damage and leads to other age-related disorders. Eliminating inflammation could be a viable anti-aging strategy since it has been identified as an endogenous component in aging. Lately, products of natural origin have been gaining attention in combating age-related diseases and chronic inflammation. Various *in vitro*, *in vivo,* and clinical studies have well documented the role of *Withania somnifera* (WS) in alleviating inflammation and aging in cells by mediating several cellular signaling pathways. This review discusses the features and mechanisms of inflammation and aging (inflammaging) and the modulation of key associated pathways by WS, examining its role as a promising candidate for future strategies against inflammaging.

**Graphical Abstract:**

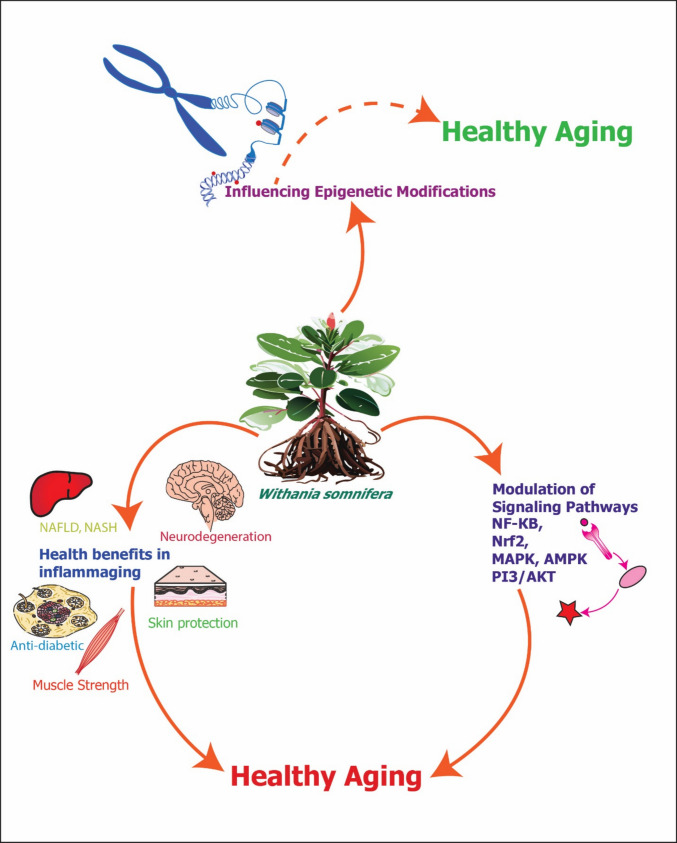

## Introduction

Aging is a universal biological phenomenon featured by a gradual decline in physiological function and resilience, predisposing individuals to a spectrum of age-associated disorders ranging from neurodegeneration to cardiovascular ailments [1]. At the molecular level, aging is linked with alterations in cellular signaling pathways, genomic instability and dysregulation of epigenetic mechanisms [2]. Notably, the phenomenon of inflammaging, a chronic low-grade inflammation state detected in aging organisms, exacerbates age-associated pathologies by promoting an environment that encourages the growth of diseases by increasing inflammation [3]. In parallel, epigenetic mechanisms contribute in the modulation of gene expression throughout the lifespan, exerting profound effects on cellular function and organismal health [4]. Epigenetic modifications viz*.* non-coding RNA-mediated regulation, DNA methylation, and histone modifications serve as a molecular interface through which environmental factors and lifestyle choices influence gene expression profiles, thereby shaping the trajectory of aging and disease susceptibility. The complex interaction between aging, inflammation, and epigenetics represents a multifaceted nexus that profoundly influences human health and disease [5, 6]. Therefore, gaining a comprehensive understanding of the complex molecular processes that contribute to inflammaging is crucial towards developing effective strategies to promote healthy aging and reducing age-related illnesses.

*Withania somnifera (*WS*)*, commonly known as Ashwagandha, holds significant promise as a therapeutic agent in mitigating age-related ailments, inflammation-driven pathologies, and epigenetic dysregulations. WS has a rich history in traditional Ayurvedic medicine, being well recognized for its anti-aging properties and therapeutic benefits in ameliorating inflammation-associated pathologies [7, 8]. Previous studies have provided compelling evidence supporting the antioxidant [9], anti-inflammatory [7] and immunomodulatory effects of WS [10], underscoring its potential as a natural remedy for promoting healthy aging and mitigating age-related morbidities [11]. Moreover, emerging research has unveiled the ability of WS to modulate epigenetic mechanisms [12], offering novel insights into its mode of action and therapeutic potential.

Although the hallmarks of aging have been elucidated previously [13, 14], there has been considerable discourse on longevity biomarkers and the most appropriate targets to reverse aging. This represents an ongoing field of research that is constantly evolving, especially given the complexity of cross-talk between multiple molecular markers as well as the putatively opposite roles played by biomarkers in different cells and tissues subtypes. This begets the question whether an intervention that targets a specific pathway is safe and non-toxic, as well as satisfies the necessary and sufficient conditions for anti-aging benefits. In this review, we aim to elucidate the relationships between aging, inflammation, and epigenetic modifications, including WS-mediated signaling pathways and its health benefits. By critically examining and integrating findings from the existing literature, we seek to clarify the molecular mechanisms underpinning the therapeutic effects of WS in aging-related pathologies, inflammation-driven diseases and epigenetic regulation. Furthermore, we endeavor to explore the effects of WS on specific organ systems affected by inflammaging, paving the way for the development of innovative strategies for promoting healthy aging and combating age-related diseases.

## Aging and Inflammation-Related Diseases

Aging is linked with deterioration of biological systems, driven by alarming risk of chronic inflammation and related diseases. Aging is often accompanied by age-linked diseases like Alzheimer’s, Parkinson’s, dementia, diabetes and cardiovascular diseases (CVD). Understanding the complex relationship between inflammation and aging is essential for discovery of new therapeutic targets and can substantially contribute to advancement in preventive and management strategies of age-related diseases as well as promotion of healthy aging behaviors.

### Neurodegenerative Diseases

Neurodegenerative diseases (NDs) are complex disorders marked by progressive neuronal degeneration [15, 16]. Aging triggers biological changes that compromise intercellular communication, telomere length and protein regulation, accumulating misfolded proteins. Moreover, mitochondrial dysfunction due to oxidative stress stimulates changes in mitochondrial DNA impairment, energy production and further increases reactive oxygen species (ROS) [17]. Cellular senescence is a common feature of aging and NDs. Senescent cells build up in the brain, resulting in persistent inflammation, and mitochondrial failure. These factors synergistically enhance ROS production resulting in cellular dysfunction, thus contributing to increased susceptibility to NDs with age progression [18]. Disruption of calcium-activated protein kinase crucial for cellular homeostasis has been reported in NDs. Further, calcium/calmodulin-dependent kinase II, a key mediator, is also associated with cytoskeletal abnormalities in NDs and aging [19].

#### Alzheimer's Disease

Age is the leading risk element in Alzheimer's disease (AD), primarily affecting older adults and is illustrated by gradual cognitive decline along with memory loss [20]. The disease is often characterized by a pathological build-up of tau tangles and amyloid-β plaques in the brain, causing neuronal dysfunction and cognitive decline [21]. Age progression accelerates AD pathogenesis through chronic inflammation, impaired protein clearance and mitochondrial dysfunction [22].

Neuroinflammation is the prime culprit in the progression of age-related AD, contributing to neuronal damage and formation of insoluble A*β* peptides. Although several functions of proinflammatory cytokines contribute to neuroprotection, the induction of proinflammatory signals result in the discharge of immune mediators, affecting neuron function and eventually causing cell death. Various pathways like nuclear factor kappa B (NF-κB), p38 mitogen-activated protein kinase (p38 MAPK), Akt/mTOR, caspase and cyclooxygenase (COX) are known to stimulate immune cells in the brain to secrete inflammatory cytokines while further contributing to AD pathology [23].

Neuroprotection in normal aging requires repressor element 1-silencing transcription factor (REST) in normal neurons, which is disrupted in AD. REST enhances stress response genes, protects against oxidative stress and amyloid β-protein toxicity, and promotes genes linked to cell survival, thereby safeguarding neurons. Deleting REST in mice has been shown to trigger age-related neurodegeneration [24].

Moreover, in AD, the lower concentration of nicotinamide adenine dinucleotide (NAD^+^) triggers cGAS-STING (cyclic GMP-AMP synthase—stimulator of interferon genes) activation, contributing to AD neuroinflammation and cellular senescence. NAD^+^ plays a crucial role in cellular functions of mitophagy and DNA repair [25]. Additionally, TREM2 (triggering receptor expressed on myeloid cells 2) plays a pivotal role in AD pathogenesis. Elevated levels of TREM2 in rats with Alzheimer-like conditions improved cognition and reduced inflammatory responses. TREM2 operates through the Phosphatidylinositol 3-kinase (PI3K)/AKT/ Forkhead box O3a (FOXO3a) pathway, offering neuroprotection by suppressing inflammation and preserving cognitive function [26]. Furthermore, systemic inflammation exacerbated by early and significant increase in tumor necrosis factor α (TNF-α), interleukins (IL and IL-1β), and cytokines in periodontal infections increases the risk for AD and cognitive impairment [27]. Therefore, therapeutic strategies aimed at modulating TREM2 may represent a promising avenue for mitigating AD pathology. Loss of function mutations in TREM2 impair microglial responsiveness, exacerbate plaque accumulation and also accelerate neurodegeneration. A precise understanding of TREM2 signaling dynamics across the disease stages remains essential before therapeutic translation.

#### Parkinson's Disease

Parkinson's disease (PD) is majorly caused by oxidative stress linked to aging, dysfunctional mitochondria, unbalanced intracellular calcium concentration, neuroinflammation, and protein aggregation, particularly α-synuclein [28, 29]. Furthermore, chronic low-grade inflammation from aging has been known to activate microglia and accelerate neurodegeneration [30]. Genetic factors including mutations in the leucine-rich repeat kinase 2** (**LRRK2) gene and environmental exposure also affect the inflammatory pathways associated with PD [31, 32]. The degeneration of dopaminergic neurons in the substantia nigra and the build-up of protein inclusions known as Lewy bodies, are the hallmarks of PD [33].

PD patients have consistently shown enhanced concentrations of pro-inflammatory cytokines. Mutations in two ubiquitin ligases, Parkin and phosphatase and tensin homologue-induced kinase 1 (PINK1), critical for preserving mitochondrial activity and protein degradation pathways, have been associated with PD. These mutations augment the pathophysiology of PD by disrupting cellular homeostasis and promote neurodegeneration [34].

Per research on the ROS network and oxidative stress in PD, intake of antioxidants and caffeine have shown promising outcomes in reducing oxidative stress by targeting pathways linked to oxidative damage and mitochondrial dysfunction [35]. Studies have identified key molecular processes, such as p62 metabolism, Kelch-like ECH-associated protein 1 (KEAP1) degradation, and mitochondrial synthesis which regulate aging and neurodegeneration in PD. Moreover, dysfunction of dopaminergic neurons in the substantia nigra is closely linked to calcium dysregulation mediated by L-type calcium channels (LTCCs), particularly the Ca_v_1.2 and Ca_v_1.3 variants [36]. Gaining an in-depth understanding of these mechanisms is crucial for developing novel targeted therapies to preserve neuronal function in PD.

#### Dementia

The World Health Organization (WHO) and the National Institute on Aging have identified tau tangles and amyloid-β plaques as the two major protein abnormalities linked to dementia, which primarily affects individuals over 65 years of age. Alpha-synuclein protein aggregates are indicative of Lewy body dementia, while TAR DNA-binding protein 43 (TDP-43) protein accumulation is linked to frontotemporal dementia. Genetic factors like mutations in Amyloid Precursor Protein (APP), Presenilin-1 (PSEN1), Presenilin-2 (PSEN2), and apolipoprotein ε4 (APOE ε4) allele also increase the risk of dementia [37].

Studies have suggested that people with vascular dementia and AD tend to have higher levels of cytokines such as IL-15, -17A, -2, -5, -12p40, -16, -1β, -6, -8 and TNF-α in their brains [38]. Individuals with enhanced chronic inflammation or anemia are at a higher risk of dementia and cognitive decline [39]. Investigations into the relationships between peripheral inflammatory markers, brain architecture, metabolism, and behavioral variant frontotemporal dementia (bvFTD) showed a higher amount of Tumor Necrosis Factor (Ligand) Superfamily Member 12 (TNFSF12), sCD30, TNFSF/BAFF, IL-2, IL-12p70, IL-17A, and TNFSF in the plasma of bvFTD patients. Further, atrophy and inflammation are most strongly correlated in the frontal-limbic-striatal brain regions of byFTD patients [40]. Dementia has also been associated with gut microbiome composition modifications, elevated concentrations of gut permeability biomarkers and inflammation [41].

### Diabetes

Diabetes shows increased occurrence with advancing age, leading to inflammation involving several key proteins [42]. The increased concentration of pro-inflammatory cytokines contributes to chronic systemic inflammation, exacerbating insulin resistance along with β-cell dysfunction. In diabetes, inflammatory biomarker C reactive protein (CRP) serves as a critical risk factor by influencing tissue fibrosis via transforming growth factor-beta (TGF-β/Smad) signaling and inflammation via NF-κB and mTOR signaling. CRP inhibits cell regeneration and accelerates aging by modulating the p21/p27-Smad3 dependent pathway [43]. Additionally, aging enhances diabetic complications by further compromising mitochondrial integrity [44].

Chondrocytes isolated from diabetes-induced osteoarthritic Sprague Dawley (SD) rats exhibited an increase protein expression of p21, p53, collagen type I alpha 1 (COL-II), and Matrix Metalloproteinase-13 (MMP-13). Therefore, prompting a higher glucose concentration can lead to cellular degradation, aging and damage [45].

### Cardiovascular diseases

The risk of CVD is increased by age-related changes in proteins that enhance oxidative stress, endothelial dysfunction and inflammation [46]. Cardiac aging involves slow and gradual cardiac dysfunction due to enhanced exposure to stress, cardiovascular illness, and mortality in elderly people. Pro-inflammatory proteins promote chronic low-grade inflammation, and inflammaging is linked to elevated levels of these proteins [47]. Enhanced amounts of circulating pro-inflammatory factors are closely linked with cardiovascular pathologies, myocardial infarction and coronary heart disease [48]. Current research is focusing on the influence of biological sex in inflammaging-related CVD. Cardiac aging in older females is facilitated by lower levels of estrogen which activates inflammatory pathways. This leads to a decrease in antioxidative defense and mitochondrial biogenesis, thus affecting overall functioning of the heart [49]. In addition to proinflammatory cytokines, an enhanced level of pro-inflammatory macrophages is observed in the myocardium of healthy older women [50].

The enhanced production of ROS and suppressed nitric oxide (NO) activity in aging cardiomyocytes and endothelial cells enhances the risk of CVD [51]. Oxidative damage is also promoted by the downregulation of Sarco/Endoplasmic Reticulum Calcium ATPase 2 (SERCA2) and the involvement of NADPH oxidase complex [52]. CVD-associated inflammaging is triggered via Apoptosis Signal-regulating Kinase 1 (ASK1-signalosome, NLR family pyrin domain containing 3 (NLRP3) inflammasome, and p38 MAPK pathways, leading to tissue vulnerability and senescence. Age diminishes angiogenic processes and the endothelial capacity to dilate, resulting in inadequate tissue perfusion and functional deterioration [53]. ROS generated by stress reactions and mitochondrial dysfunction increase oxidative damage, further aggravating inflammation and several cardiovascular anomalies [54].

Matrix metalloproteinases (MMPs) comprising varied zinc-dependent endopeptidases degrade elastin, collagen and other extracellular commodities upon stimulation from inflammatory signals. A proinflammatory milieu is formed by breakdown of vasodilators and vasoconstrictors by MMP which modifies vascular smooth muscle cells and endothelial cells to express proliferative, senescent, migratory, and secretory phenotypes. This results in arterial remodeling, calcification, decreased vasodilation, fibrosis, and stiffness [55].

### Muscle

Age-related changes in protein metabolism result in a loss of muscle mass and strength. These changes are indicated by a decrease in key proteins like actin and myosin vital for muscle contraction. An increase in catabolic activity combined with a decrease in anabolic cues like testosterone and growth hormones leads to muscle deterioration. Moreover, mitochondrial dysfunction and oxidative stress also impair protein synthesis and muscle repair. Senescent cells are a crucial component of the skeletal muscle regenerative niche. Senescence prevents lifelong regeneration by promoting an arthritic environment and inflammation. However, muscle regeneration is accelerated by senescent cells when targeted by reduction or CD36-mediated inflammatory regulation [36]. Elevated glutamine synthetase (GS) activity and lowered glutamine levels are associated with aging. Upon muscle injury to prevent glutamine shortage, macrophages maintain high levels of GS in the absence of glutamate dehydrogenase 1 (GLUD1). Glutamine from macrophages is known to trigger mTOR and thereby promote cell division in satellite cells by SLC1A5 (amino-acid transporter). Thus, targeting SLC1A5 or GS promotes muscle regeneration, whereas inhibiting macrophage GLUD1 promotes muscle rejuvenation [56]. Aging causes weakened ocular atrophy 1 (OPA1) and intensifies muscle loss by decrease in the OPA1 protein level. In adult mice, loss of OPA1 resulted in rapid aging followed by death, however the DNA remained unaltered. Furthermore, a stressed endoplasmic reticulum results in aging, muscle loss and mitochondrial dysfunction. On the contrary, inhibiting fibroblast growth factor (FGF21) or blockage of ER stress inhibits muscle deterioration and aging. Mitochondrial dysfunction in muscles can also initiate an ER-dependent signal cascade that affects metabolism and aging in general [57].

### Liver

Increased levels of proinflammatory cytokines influence the advancement of liver diseases in older people. The subsequent inflammatory responses influence metabolic dysfunction, oxidative stress, and gut microbial translocation, leading to liver fibrosis and impaired hepatic function [58]. Hepatic regenerative capacity decreases with age due to functional changes in CCAAT/enhancer-binding protein alpha (CEBPα) which forms an age-specific complex with Rb and E2F4, thereby suppressing E2F-dependent gene expression vital for liver growth [59]. Moreover, deficiency of IGF2 has also been demonstrated to suppress mitochondrial function, raise CCAAT/ CEBPB levels, and prevent senescence phenotypes via CCAAT/enhancer-binding protein beta (CEBPB) inhibition [60].

Liver aging involves enhanced necroptosis contributing to chronic inflammation, potentially leading to hepatic fibrosis and chronic liver disease. Hepatocellular carcinoma amplifies inflammation in liver aging through necroptosis as a result of DAMP release. Reportedly, aged mice show significant phosphorylated necroptosis markers mixed lineage kinase domain-like protein (MLKL) and receptor-interacting serine/threonine-protein kinase (RIPK3/RIPK1), increased inflammation, fibrosis, and elevated M1 macrophage markers. Aged individuals exhibit higher levels of TNFα, IL6, and IL1β inflammatory markers in the liver. However, the inhibition of necroptosis through necrostatin-1 s (Nec-1 s) injections over a short period effectively mitigates these effects [61].

### Skin

Aging skin exhibits structural changes and increased inflammation due to protein modifications. These are direct results of the degradation of collagen and elastin structures, critical for maintenance and function of the skin [62]. Reduced production of collagen and damaged collagen fibers are major causes for wrinkles and decreased firmness of the skin. Moreover, elastin damage is the foundation for reduced skin elasticity and sagging. The deposition of advanced glycation end products (AGEs) is further known to damage skin proteins and promote inflammation. Research shows the ability of aged skin to fight and heal against infections is hampered due to a decrease in Langerhans cells, the first line of defense against pathogens [63].

Induced DNA damage by UV-B radiation disrupts the equilibrium of the extracellular matrix by triggering NF-κB and p38MAPK pathways and releasing IL-1β,IL-6, and TNF-α [62]. Moreover, immunosuppression and chronic inflammation contribute to disruption of tissue homeostasis by secondary degeneration in inflammatory tissues and an increase in senescent cells. The degenerative changes are triggered by ROS/RNS, anti-inflammatory cytokines, and increased catabolism of L-arginine and L-tryptophan through the activation of Arginase 1 (ARG1) and indoleamine 2,3-dioxygenase 1 (IDO1). Cytokine IL-10 decreases inflammation by disrupting autophagy and antigen presentation, upsetting the protein homeostasis in the inflammatory tissues [64].

Induced UV radiation demonstrates an enhanced TGF-β-mediated suppressed cell proliferation, and tissue fibrosis along with activation of MMPs and collagenases [65–67]. Moreover, TGF-β signaling displays altered chromatin architecture, cellular senescence and improved cardiac aging, making it an important asset in rejuvenation studies [68]. Photoaging features breakdown by TGF-β of collagen and elastin fibers preventing keratinocyte proliferation [66]. Immunosuppressive cells such as myeloid-derived suppressor cells (MDSCs) secrete ROS-inhibiting TCR-mediated T cell activators and also initiate latent TGF-β to produce anti-inflammatory and pathological responses [69, 70].

## WS and Signal Transduction

Despite the various pathologies associated with inflammaging, nutraceuticals and dietary supplements are gaining attention due to their supportive health attributes. WS has been used since ancient times in traditional Indian Ayurvedic medicine owing to its beneficial health effects.

### Immunomodulation

WS is a natural, herbal immunostimulant which improves health and prevents illness in individuals with few side effects [71–73]. Various *in vitro*, *in vivo* and clinical reports show evidence supporting the role of WS in immunomodulatory activities; it significantly improves immunity in human subjects [74, 75]. The immunostimulatory activity of WS has been attributed to the occurrence of bioactive compounds like steroids (withanolides), flavonoids and lactones [76].

A randomized, placebo-controlled, double-blind study of a WS extract was performed by Tharakan *et al.* for an initial period of 30 days. It revealed a significant rise in Ig, T-cells, B-cells, natural killer cells (TBNK) cells and cytokines (IFN-γ, IL4). While the placebo group showed a significant decline in TBNK cells, the cytokine and Ig levels did not exhibit any significant change. Doubling the study duration to 60 days with a crossover design (placebo and WS) revealed a significant increase in TBNK cells, cytokine, and Ig levels while subjects with continued WS supplementation showed significant improvements as well. The authors concluded that WS modulates both innate and adaptive immune systems, improving the overall immune profile of healthy subjects [75].

Saminathan *et al.* [77] performed a case- control study to correlate inflammatory markers in arthritis on 35 subjects. The study demonstrated an increased TNF-α and lowered IL-10 levels in the test subjects. An oral supplementation of 300 mg/kg was reported to significantly lower the TNF-α, IL-1β, IL-6 proinflammatory cytokines in collagen-induced arthritic (CIA) rats [74]. Immunomodulation of WS is suspected to be mediated via increased IL-10 secretion and inhibition of NF-κB. However, the high animal dose (300 mg/kg) raises questions about an appropriate equivalent dosing and pharmacokinetic translation in humans. Additionally, current research focuses mainly on selected inflammatory cytokines without exploring broader immune cell responses or long-term clinical outcomes.

The aqueous and ethanolic extracts of WS exhibiting withanolides significantly enhanced cell mediated immune response and triggered B- and T-cell proliferation along with the Th1 response in immunocompromised mice [78, 79]. Additionally, a herbal blend of WS also improved cell-mediated immune response with an increased CD4^+^ and CD8^+^ T-cells in test chicks compared to the control when assessed for viral diseases [80]. However, aqueous and ethanolic WS leaf and root extracts at doses of 50 mg/kg and 100 mg/kg demonstrate a decreased leukocyte count in guinea pigs infected with *E. coli* contrary to its immunostimulatory effects [81]. Rohu fishes exhibit an enhanced survival rate along with improved phagocytic and lysosomal activity of immune cells after bacterial infection when supplemented with WS root powder for 42 days [82]. Chandran & Patwardhan, in their *in-silico* network ethnopharmacological study highlighted the potential of bioactive compounds of WS in the modulation of immune pathways. However, a suitable model for validation could be incorporated into the study [83]. WS possesses immunomodulatory properties through effectively suppressing the levels of type-2 cytokines, type-2 inflammation markers, TNF-α and IgE, while also triggering proliferation of splenocytes [84, 85]. The Th1-modulating properties of WS have been reported to significantly decrease the concentration of CD38-expressing CD8^+^ T lymphocytes in human immunodeficiency virus (HIV) patients. The immune response, however, varies with differing conditions in HIV patients. The foundations of the potential of WS against viral infections could thus be established if conducted with a larger cohort [86, 87].

Immunostimulant effects of WS in COVID-19 have been well-documented. Studies have reported that enhanced serum level of Granulocyte–Macrophage Colony-Stimulating Factor (GM-CSF) and binding of GM-CSF with its receptors resulted in stimulation of cytokines in COVID-19 patients. Molecular docking indicates a significant binding affinity of withanolide A (WA) with the GM-CSF receptor, uncovering a potential new path for the development of new therapeutic approaches targeting this receptor to relieve cytokine storms [84, 88].

Research has not yet been conducted on how WS affects human immunomodulation at the cellular level. However, the literature on immunoregulatory cellular responses by WS lays new foundations in immunotherapy.

### WS in Aging and Inflammation

WS is known to play an important role in aging via inhibiting various signaling pathways. Withagenin A diglucoside (WAD) has been demonstrated to inhibit pro-inflammatory cytokine expression, decelerating skin aging. Collagen cross-linking is at the epicenter for collagenous tissues dysfunction. Aging also influences hyperglycemia—affecting renal, retinal and cardiovascular tissues—contributing to severe illness and death [89]. WA offers stress tolerance and extends life span through mediating the insulin signaling pathway in *Caenorhabditis elegans* [90]. Although WAD and WA exhibit properties extending life span and myoblast differentiation, these findings remain largely preclinical. A formulation from WS, *Silybum marianum* and *Trigonella foenum-graecum* revealed an upregulated Akt and p38 MAPK/myogenin-dependent myoblast differentiation in C2C12 myoblasts contributing to muscle physiology in mammals [91]. Therapeutic roles of WS root powder and ethanolic extract were compared with metformin for glucose-mediated collagen glycation and cross-linking. The study revealed the activity of WS to be comparable with metformin and ethanolic extract to be more potent than WS root powder. Further, tendon collagen incubated with glucose increased glycation, AGE and collagen cross-linking when treated with WS and metformin in adjuvant-induced arthritic rats [92]. However, the underlying mechanisms of these anti-glycation properties of WS are yet to be elucidated.

Oral administration of WS for 6 months (500 mg capsules twice a day) resulted in a significant drop in the level of malondialdehyde (MDA) and increase in superoxide dismutase (SOD). This contributes to countering ROS-mediated injury and preventing oxidative stress in healthy human subjects [93]. Nuclear factor erythroid 2-related factor 2 (Nrf2), a cytoprotective against oxidative stress, shows decreased expression levels with age. An *in vitro* study demonstrated that a combination of *Rosmarinus officinalis*, WS and *Sophora japonica* triggered Nrf2 activation [94]. A study on *Drosophila melanogaster* using aqueous and ethanolic extracts of WS showed improved stress-induced behavioral changes with the aqueous extract being more effective than the ethanolic [95].

WS exhibits anti-inflammatory activity through mediating various signaling pathways and upregulating anti-inflammatory markers. Interestingly, hot water extract of boiled WS leaves and roots were examined for skin inflammation on HaCaT human keratinocyte cell line. The results depicted suppressed mRNA expression for inflammatory cytokines and upregulated levels of anti-inflammatory cytokine TGF-β1 *in vitro* and *in vivo*.

These anti-inflammatory properties were attributed to WS’s ability to inhibit NF-κB and MAPK pathways and to modify cytokine expression [96] (Table I). The synergistic effect of leaves of WS and dried stem of *Tinospora cordifolia* also alleviated anxiety-linked behavior and related inflammation. The mode of action appears to be via modulating key inflammatory markers and stress response in middle-aged female rats [97].

**Table I:** Health Benefits of *Withania somnifera* in Inflammaging and Associated Diseases

Health Benefits	Source	Type of Study	Mode of Action	Functional Outcome	References
Immunomodulation	WS root and leaf extracts	Randomized, placebo-controlled, double-blind study	Increases Ig’s, cytokines (IFN-γ, IL4) and TBNK cells	Improves innate and adaptive immune systems	Tharakan *et al.*, [75]
	Withanolide A	Rat Model	Increases the expression of IL-2 and IFN-gamma	Triggers B and T cells proliferation along with Th1 response	Kour *et al.*, [102]
	WS root extract	*In vitro* and *in silico*	Lowers the type 2 cytokines levels, type 2 inflammation markers, TNF-α and IgE	Enhances cell-mediated immune response with CD4 + and CD8 + T cells	Chandran *et al.*, [83] Dhawan *et al.*, [86]
Anti-neuroinflammation	Withaferin A	*In vitro*	Production of TNFα, COX-2, and iNOS via LPS/TLR4 pathway	Inhibition of NF-κB activity	Martorana *et al.* 2015 [109] Heyninck *et al.*, [110] Atluri *et al.*, [112]
Improved Cognitive Function	Withanone	Rat model	Decreases concentrations of proinflammatory cytokines and lipid peroxidation	Inhibition of amyloid β-42	Pandey *et al.,* [111]
Alzheimer’s Disease	WS root extract	*In vitro*	Increase in expression of the Na⁺–Ca^2^⁺ exchanger	Mitigates amyloid toxicity	Kumar *et al.*, [116]
	Withaferin A from fruit	*In vitro*	Blocks β-amyloid active sites and inhibits fibril formation	Mitigates amyloid toxicity	Jayaprakasam *et al.*, [119]
	WS root extract	APP mice model	Upregulates LRP and suppresses cholinesterase activity	Reverses Aβ accumulation and toxicity	Sehgal *et al.*, [115] Kurapati *et al.*, [117]
Parkinson’s Disease	WS root extract	Mouse Model	Inhibits expression of iNOS and Bax, induces Bcl-2	Improves physiological abnormalities	Prakash *et al.*, [124] Rajashankar *et al.*, [125]
Cardioprotection	WS root extract	Rat Model	Increase Glutathione, CAT, SOD, LDH activities and suppresses MDA level	Increases endogenous antioxidants	Mohanty *et al.,* [129]
	Withaferin A	Rat Model	Upregulation of AMPK levels and suppress casapase-9	Prevents Myocardial Ischemia	Guo *et al.*, [130]
	WS extract	*In vitro*	Nrf-2 activation and stimulation of phase-II detoxifying enzymes	Increase antioxidant activity	Reuland *et al.*, [131]
Arthritis	WS root extract	Rat model	Increases secretion of IL-10 and supresses NF-κB activity	Lowers the pro inflammatory cytokines level	Khan *et al.*, [74]
Anti-inflammatory	WS root and leaf extracts	*In vitro*	Inhibits NF-κB and MAPK pathways and alters cytokine expression	Supresses mRNA expression for inflammatory cytokines	Sikandan *et al.*, [96]
Anti-aging	WS root extract	*In vitro*	Prevents collagen modification enzymatic glycation and cross-linking	Hinders glycation induced pathogenesis	Babu *et al.*, [92]
Antioxidant	WS root extract	Mouse model	Increases GSH level and stimulates SOD, CAT activities	Inhibits lipid peroxide production	Kumar *et al.*, [116]
	WS extract	*In vitro*	Upregulates FOXO3A and SIRT3	Enhances longevity and healthy aging	Pradhan *et al.*, [105]
	Withaferin A	Mouse model	Upregulates HO-1, Prdx-1, and SOD-2 via Akt pathway	Prevents peroxide-induced apoptosis and enhances cell survival	Yan *et al.*, [107]
Anti-diabetic	WS root and leaf extracts	Rat Model	Intervening the NRF2/NF-κB signaling pathway	Reduces glucose level and improves lipidemic profile	Udayakumar *et al.* [136] Kyathanahalli *et al.*, [138]
Muscle Supplement	WS root extract	Randomized, double-blinded, placebo-controlled trial	Possibly through elevated testosterone levels	Improvements in cardiorespiratory fitness, muscle strength, recovery, and overall physical fitness	Wankhede *et al.*, [142] Choudhary *et al.*, [144]

### WS in Oxidative Stress

WS root extract shows significant antioxidant potential. An *in vivo* assessment in paraquat-induced oxidative stress mice model revealed suppressed lipid peroxide production, decreased glutathione (GSH) levels along with altered the SOD and catalase (CAT) activities in a dose-dependent approach [98, 99] (Table I). Interestingly, in patients with ischemic stroke, supplementation of WS eased middle cerebral artery occlusion induced stress, cognitive impairment, mitochondrial dysfunction and apoptosis [99]. Aqueous and hydroalcoholic extracts of WS have been suggested to reduce oxidative stress, improve fitness and sleep in *Drosophila* [95, 100]. A randomized, double-blind, placebo-controlled study on 125 human subjects indicates that an intake of WS root extract sustained release capsule (300 mg) for 90-days increases memory, attention, sleep quality, overall well-being and reduce stress [101]. $\kappa$ An aqueous leaf extract of WS was investigated in female Wistar rats for alleviating the effects of acute sleep deprivation on immune function. The study revealed an inhibition in stress-induced apoptosis as well as increased levels of NF-кB, AP-1, Bcl-xL and cytochrome c expressions. However, an enhanced concentration of pro-inflammatory cytokines was also observed [102]. Remenapp *et al.* [103] report that a WS intake of 225 mg/day improves cognitive function, particularly attention and working memory, and reduces symptoms of anxiety and stress in healthy human subjects. The findings of various studies on the adaptogenic properties of WS suggest it to be a potential natural supplement to improve cognitive function, reduce symptoms of oxidative stress and anxiety, and enhance longevity [104].

Pradhan *et al.* [105] examined the oxidative effect of WS on longevity and healthy aging genes i.e., FOXO3A and SIRT3 on 473 subjects of 3-age groups viz. young: 20–30 years, old: 60–79 years, and oldest: > 80 years (Table I). The study highlights decreased concentrations of FOXO3A and SIRT3 with age, thus making them useful markers for aging. Further *in vitro* assessment on HEK cell lines demonstrated that WS countered age-related decline by upregulation of Forkhead box O3a (FOXO3A) and Sirtuin 3 (SIRT3) proteins. However, the mechanistic pathway by which WS modulates these longevity-associated genes remain unclear.

Withaferin A from WS is known to induce Nrf2, which partakes in the oxidative stress response via PTEN/PI3K/AKT pathway activation. The activation of Nrf2 leads to a downstream production of antioxidant enzymes like CAT, SOD, GSH synthase, peroxidases, reductases and antioxidant proteins. The bioactive compounds in WS are recognized for stabilizing oxidative conditions and may prevent oxidative stress-associated diseases [106, 107].

### WS in Neurodegenerative Disorders

#### Anti-neuroinflammation

In AD, the microglia and phagocytes of nervous system are triggered by soluble A*β* oligomers and A*β* fibrils binding to cell surface receptors, inducing inflammatory responses via activation of LRR, NOD, NLRP3, and NF-κB. The activated NLRP3 and NF-κB inhibit A*β* phagocytosis by microglia, enhancing A*β* fibrils accumulation and leading to neuroinflammation [108].

*In vitro* studies have shown that withaferin A inhibits NF-κB activation, attenuating its associated pathways and the subsequent production of TNFα, COX-2, and iNOS triggered by the LPS/TLR4 pathway stimulation in astrocytes [109, 110]. *In vivo* investigations into a WS root extract zeroed in on withanone to be the most effective compound at a dosage of 20 mg/kg, given orally and once daily to Wistar rats for 21 days. This regimen improved cognitive function and inhibited amyloid β-42 and lipid peroxidation, in addition to reduced levels of pro-inflammatory cytokines [111]. Atluri *et al.* [112] investigated the anti-inflammatory properties of withaferin A from a WS root extract on SH-APP cells and microglial cell lines at varying concentrations for 48 h. The study concluded withaferin A inhibits the NF-κB and RELA transcription factors, while upregulating inhibitor of nuclear factor kappa-B kinase (IKBKB and IKBKG), and downregulating JUN and STAT genes. However, there is a substantial need for mechanistic investigations and controlled clinical trials to substantiate the therapeutic efficacy of WS in ND.

#### Alzheimer’s Disease

At present, the two main symptomatic treatments of AD include: acetylcholine esterase inhibitors—which enhance the availability of acetylcholine at synapse—and non-competitive NMDA receptor antagonists, which mediate calcium channel opening and decrease glutamate neurotoxicity [113]. WS treatment alleviated behavioral and cognitive defects triggered by Bisphenol A, an endocrine disruptor in Swiss albino mice by restoring NMDA receptors [114]. Interestingly, an oral treatment schedule with WS root extract for 30 days in AD-induced transgenic mice, reversed Aβ accumulation and behavioral deficits by upregulating low-density lipoprotein receptor-related protein [115]. WS root extract has also shown neuroprotective properties in AD patients against the cytotoxic effects of Aβ (1–42) and H_2_O_2_ in PC-12 cells [116].

WS has been reported to interfere with the amyloid induced toxicity in SK-N-MC neuronal cells, thereby reversing amyloid-induced reduction in spine density, area, and length. Additionally WS supplementation suppressed cholinesterase activity and reduced Aβ internalization [117]. Bioinformatics studies by Grover *et al.* [118] revealed that WA binds to different residues of the acetylcholinesterase enzyme and could modulate its functions. Withaferin A and C treatment was found to obstruct cell death in PC-12 by amyloid toxicity through blocking β-amyloid active sites and inhibiting fibril formation [119]. Moreover, intake of a 250 mg WS extract capsule twice/day for 2 weeks, enhanced memory and cognitive performance through the modification of cholinergic neurotransmission in healthy human subjects [120]. Nitti *et al.* [121] in their study suggested that WA mitigates AD by elevating the expression of heme oxygenase 1, a protein involved in neuroprotection.

#### Parkinson’s Disease

PD is highlighted by the degeneration of dopaminergic neurons from the substantia nigra pars compacta. It is linked with Lewy bodies, composed of insoluble aggregates of alpha-synuclein [108, 122]. The impaired defense mechanism, enhanced production of ROS, and dysregulation of antioxidant enzymes like CAT, SOD, and GPX leads to abundance of free radicals and consequently, neuronal damage [123]. An ethanolic WS root extract (100 mg/kg for 9 weeks) has been proven to reinforce nigrostriatal neuroprotection and enhance motor function in PD mouse models. WS, through the reduction of iNOS expression and anti-apoptotic proteins, was found to deter oxidative damage [124]. Treatment with WS for 7 or 28 days (100 mg/kg) in 1-methyl-4-phenyl-1,2,3,6-tetrahydropyridine (MPTP) PD model showed an elevated DA, HVA, and DOPAC expression in comparison to the control. This extends the scope of WS to healing oxidative and physiological abnormalities caused by PD [125]. Standard PD drugs often cause dyskinesis over time. WS could potentially lower the required dose and also minimize side effects.

### Cardioprotection

Age-related diseases have been linked with various components viz., enhanced oxidative stress, inflammation, apoptosis, vascular and myocardial deterioration. Additionally, the possibility for other illnesses like frailty, obesity and diabetes is also greatly enhanced [126, 127]. The cardioprotective and cardiotonic properties of WS have been cited in ayurvedic text and used in traditional medicine for relieving CVD [128].

WS treatment at 50 mg/kg showed a substantial increase in activities of glutathione, CAT, SOD, lactate dehydrogenase with a decrease in lipid peroxidation marker MDA level in comparison to the control group. The cardioprotective activity of WS may be attributed to increased endogenous antioxidants, maintenance of myocardial antioxidants and altered hemodynamic parameters [129]. Withaferin A was observed to upregulate AMPK levels and halt caspase-9 activation at a dose of 1 mg/kg in wild mice, improving cardiac function; however, no such improvements were observed in transgenic mice [130]. *In vitro* studies suggest that polyherbal formulations of WS are cardioprotective by stimulating phase-II detoxifying enzymes and reduce apoptosis in an Nrf-2-dependent approach in HL-1 cardiomyocytes [128, 131]. The cardioprotective role of withaferin A was examined on human umbilical vein endothelial cells (HUVECs) regarding its associated signaling pathways. Withaferin A was demonstrated to inhibit phorbol-12-myristate 13-acetate (PMA), TNF-α, IL-1β and TNF-α-converting enzyme activity. Additionally *in vivo*, withaferin A led to a decline in p38 PMA-stimulated phosphorylation, ERK 1/2, and JNK in mice, depicting its role as a potent cardioprotectant [132]. By simultaneously activating the Nrf-2/Phase-II detox pathway and the AMPK pathway while inhibiting the MAPK inflammatory cascade, WS has a multi-faceted approach in preventing ischemic ailments.

### Antidiabetic

Diabetes has become one of the most prevalent and serious health issues among the elderly. Older people with diabetes possess high risk of several chronic diseases like myocardial infarctions, visual impairments, and end-stage renal diseases [133, 134].

Few reports pertain to the antidiabetic property of WS, although preclinical trial results are quite promising. Studies support the vital role of WS root and leaf extracts in lowering blood glucose levels *in vivo* [104, 135, 136]. Withaferin A has been demonstrated to reduce glucose level *in vivo* at a dosage of 2 and 10 mg/kg in a type 1 diabetes mellitus mice model via the NRF2/NF-κB signaling pathway [137]. Kyanthanahalli *et al.* [138] found that oral administration of WS influences diabetes-induced testicular oxidative disturbances in prepubertal rats. The rats were supplemented with STZ (streptomycin) + WS (500 mg/kg) orally for 15 days. WS stabilized the elevated ROS levels, lipid peroxidation in testis cytosol and mitochondrial function of diabetic rats. The enzyme activities of glucose-6-phosphate dehydrogenase, lactate dehydrogenase and 3-beta hydroxysteroid dehydrogenase were restored through fractional conditioning in testis of diabetic rats—suggesting a role for WS in the improvement of diabetic testicular dysfunction. The antidiabetic property of WS has been confirmed by *in silico* studies [139]. This calls upon more comprehensive clinical studies, of which very few are present, on this subject [140].

### Increased Muscle Strength

Muscle starts to deteriorate at the age of 40 and decline at the rate of 1–2% per year after 50 years. The progressive degradation of muscle mass with aging is because of slower anabolism *versus* enhanced catabolism. WS has been recognized for strengthening the weakened skeletal muscles attributed to anti-stress, adaptogenic, anti-inflammatory as well as antioxidant properties [141, 142].

Oral supplementation of WS root extract (300 mg) in 29 healthy male (aged 18–50) subjects for a period of 30 days twice daily, caused a significant increase in muscle strength along with increases in muscle mass in the arms and chest. The results were attributed to stabilization of plasma creatine kinase levels resulting in a significant lowering of exercise-induced muscle myocyte damage when compared to the placebo. Additionally, treated groups noticed a significant enhancement in testosterone levels along with reduced body fat percentage [142]. A aqueous root and leaf extract of WS (500 mg/ day) was supplemented for muscle training and recovery in a randomized double-blind model on 38 healthy male subjects. Parameters like body composition, muscular strength, power, and endurance were assessed at the commencement of the study and 12 weeks post-WS supplementation. WS intake improved body strength, proper allocation of body mass, endurance and was well tolerated in recreationally active men [143]. Another randomized, double-blind, placebo-controlled study was conducted on 50 healthy volunteers to evaluate the efficacy of WS root extract in enhancement of cardiorespiratory endurance and improvement of life quality. Cardiorespiratory endurance was evaluated in terms of oxygen intake at the highest physical exertion stages in a 20 m shuttle run test. Further quality of life was assessed by a WHO questionnaire on self-reported quality of life. The findings suggest enhancement in cardiorespiratory endurance as well as improved life quality in healthy athletic adults by intake of WS root extract [144]. Yet another randomized, double-blind, placebo-controlled study was conducted by Shenoy *et al.* [145] on forty elite Indian cyclists to evaluate the effect of WS on cardiorespiratory endurance capacity. The experimental groups were supplemented with WS root extract (500 mg capsule) for eight weeks and outcomes were measured by a basic treadmill test for aerobic capacity, metabolic equivalent, and respiratory exchange ratio. A significant improvement in the parameters was observed in the test groups supplemented with WS whereas no such effects were observed in the placebo groups. Since these clinical studies were centered around healthy male subjects, it is not evident whether the findings hold true for post-menopausal women susceptible to muscle frailty.

A study on rats equivalent to 60–65 years in human age supplemented them with different combinations of (a) standardized WS extract, (b) a protein cocktail including soybean and quinoa, and (c) a reference standard comprising of combination of WS extract and protein cocktail or whey protein for 60 days. The total protein, inflammatory markers, AMPK, MDA, antioxidant enzymes, along with apoptotic genes were evaluated at the end of the treatment. WS extract + protein treatment was found to produce the best enhancement in muscle strength, while all treatments decreased aging-elevated glucose, TNF-α, AMPK, MDA, Bax levels and considerably restored the age-depleted levels of GSH, SOD, CAT, and Bcl-2. The regaining of muscle strength and functionality was indicated by improvements in the grip strength as well as remarkable bicep mass in all the treatments. In conclusion, the WS and protein fusion restrained muscle loss and strengthened the skeletal muscle via reduction of inflammation, oxidative stress and apoptosis along with increased ATP accessibility [141].

## Role of WS in Key Signaling Pathways

### PI3K/AKT Pathway

Withaferin A inhibited the cellular proliferation and apoptosis of A549 via suppressing the activation of the PI3K/Akt pathways in non-small cell lung cancer. Withaferin A in A549 cell line suppressed cell proliferation in a dose-dependent manner; the apoptotic rate was highest at 48 h with remarkably lowered Bcl-2 level and higher Bax expression along with shrunken caspase-3 levels [146] (Table II). WS leaf extract was shown to regulate the maintenance of synaptic elasticity and prevention of cognitive decline in high fat diet rats. WS activated the PI3/Akt pathway as well as elasticity via increasing the concentration of phosphorylated Akt-1 and immediate early genes i.e., c-Jun and c-fos [147]. In pancreatic cancer, withaferin A, in combination with oxaliplatin, suppressed growth and apoptosis through mitochondrial dysfunction along with PI3K/Akt pathway inactivation *in vitro* and *in vivo* [148]. A focused study on the effect of withaferin A on apoptosis along with generation of intracellular ROS in rabbit articular chondrocytes was conducted. Withaferin A treatment led to apoptosis of chondrocytes in a dose-dependent approach via activation of PI3K/Akt and JNKinase [149]. The neuroprotective effects of withaferin A include anti-apoptotic and anti-proliferative activities through the suppression of PTEN, which further activates the PI3K/AKT/mTOR and PI3K/AKT/GSK3b pathways, as well as the inhibition of vascular smooth muscle cell migration [150]. The ethanolic extract of WS decreased sarcopenia-linked muscle atrophy in aged mice. Treatment of WS extract enhanced phosphorylation levels of the PI3K/Akt/mTOR pathway, upregulation of myogenic proteins as well as accelerated differentiation and fusion of myoblast to myofibers [151]. The hypolipidemic and anti-inflammatory properties of WS against high cholesterol diet was evaluated in Sprague–Dawley male rats. The results revealed the anti-atherosclerotic property of WS through regulation of inflammatory mediators as well as apoptosis, via an increase in PI3K/AKT signaling pathway [152]. Palliyaguru *et al.* [153] evaluated role of withaferin A in acetaminophen (APAP)-induced hepatic toxicity in mice for Nrf2 signaling and concluded that withaferin A induced Nrf-2 dependent cytoprotective enzyme expression in mice via a PTEN/PI3K/AKT-dependent approach.

**Table II.**
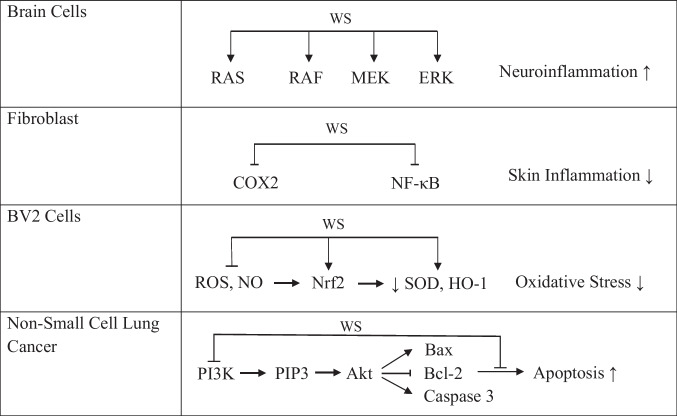
Signal Transduction Pathways Mediated by *Withania somnifera* in Inflammaging

Abbreviations: Akt: serine/threonine kinase; Bax: Bcl-2-associated protein x; Bcl-2: B-cell lymphoma; COX-2 : cyclooxygenase-2; ERK: extracellular signal-regulated kinase; MEK: mitogen‐activated protein kinase; NF-κB: nuclear factor kappa B; Nrf2: nuclear factor erythroid 2-related factor 2; NO: nitric oxide; P1P3: phosphatidylinositol (3,4,5)-trisphosphate; RAF: rapidly accelerated fibrosarcoma; RAS: reticular activating system; ROS: reactive oxygen species

### NRF2 Pathway

The antioxidants from WS improve oxidative balance, preventing oxidative stress-related diseases. Withaferin A modulates oxidative stress via regulating Nrf2, which regulates the antioxidant enzymes expression in response to an oxidative stress [106]. The anti-inflammatory and antioxidant properties of two constituents of WS viz*.* withaferin A and WA, were investigated using murine immortalized BV-2 microglial cells. WS extract effectively suppressed lipopolysaccharide-induced nitric oxide and ROS production, stimulated NRF2 pathway as well as induced heme oxygenase-1 in BV-2 cells (Table II). Further, withaferin A was ten times more effective in inhibition of lipopolysaccharide-induced NO production along with the stimulation of the NRF2/HO-1 pathway and downregulation of the NF-κB pathway [154]. In another study comparing the anti-inflammatory and antioxidant effects of botanical extracts from WS, *Sutherlandia frutescens* (SF) and *Euterpe oleracea* (EO) in the immortalized rat astrocyte (DI TNC1) cell line, the WS extract actively induced expression of Nrf2 and HO-1 in DI TNC1 astrocytes in when compared to SF and EO extracts [155].

### NF-κB Pathway

The activities of the aqueous WS root extract in skin inflammation was studied *in vitro* and *in vivo*. It possessed anti-inflammatory property through NF‑κB and MAPK pathway suppression, potentially protecting the skin from inflammation [96]. In regards to chronic inflammation and renal dysfunction, the potential of WS, *Sutherlandia* and elderberry were investigated *in vitro*. WS mediated anti-inflammatory responses by suppressing CCl2 expression in response to TNF-α stimulation, which was in turn activated by suppressed NF-κB activity [156]. Aqueous WS leaf extract was tested *in vivo* against LPS-induced neuroinflammation and related behavioral impairments, revealing protective properties. The molecular mechanism involves inhibition of NF-κB, P38, and MAPK pathways which were initiated by LPS [157]. WS crude ethanol extract was investigated for anti-inflammatory potential in peripheral blood mononuclear cells from normal persons and rheumatoid arthritis patients *in vitro*. Withaferin-A blocked NF-κB activation and inhibited production of COX-2, indicating potential use in treating a variety of inflammatory conditions in age-related diseases like inflammation linked with arthritis, cystic fibrosis and inflammatory bowel disease (Table II). WS extract, specifically withaferin A, inhibited LPS activated nitric oxide production via suppression of nuclear translocation of NF-κB, AP-1, and I*κ*B*α* phosphorylation in normal and mononuclear cells of RA patients [158]. In arthritic rats, WS aqueous root extract reduced pro-inflammatory cytokine generation by inhibition of NF-κB activity [74]. In AD, the NF-κB pathway inhibits phagocytosis of Aβ fibrils, which causes Aβ fibril accumulation and neuroinflammation [159]. Additionally, withaferin A was also reported to suppress NF-κB activation through inhibition of NF-κB phosphorylation and IκB kinase stimulation, preventing neuroinflammation [160].

### MAPK Pathway

MAPKs increase intracellular signaling to various cellular processes like cell division, proliferation, survival as well as proliferation [161]. WS upregulates MAPKs which play an important role in muscle physiology [162]. Encapsulation of WS extract in chitosan-alginate bipolymeric nanocapsules was tried in a thioacetamide-induced hepatic encephalopathy rat model. The nanocapsules resulted in improved survival, general motor activity and cognitive task-performance. Nanocapsules perform their hepatoprotective and neuroprotective roles via restoring the Nrf2 and MAPK signaling pathways in liver and brain tissues [163]. Another study highlighted the neuroprotective role of WA in glutamate-induced excitotoxicity in retinoic acid-differentiated Neuro2a neuroblastoma cells. WA significantly reduced influx of intracellular calcium along with ROS production. The protective effects of WS were due to suppression of MAPK proteins as well as P13/Akt activation [164]. The effect of withaferin A on collagen loss expression plus inflammation in rabbit articular chondrocytes was investigated. Withaferin A-treated cells were concluded to possess inhibited phosphorylation of P13/Akt, p38 and JNK, suppressing the expression of COX-2 in rabbit articular chondrocytes [165]. In skin aging, withagenin A diglucoside suppressed the inhibition of MAPK, Akt, NF-κB phosphorylation, and COX-2 expression along with inhibition of pro-inflammatory cytokines, suggesting a potential role of WS compounds in cosmetics and pharmaceuticals associated with skin aging [89].

## WS and Epigenetic Modifications

The interconnecting aspect of epigenetics and inflammaging is a crucial area of aging research. Epigenetic alterations are one of the hallmarks of aging—in which epigenetic alterations viz*.* DNA methylation, histone modification and non-coding RNA lead to modifications in DNA structure—resulting in the creation of a pro-inflammatory environment and affecting epigenetic patterns [5, 166]. Alterations like DNA methylation and histone modification result in inflammatory consequences during aging while non-coding RNAs (ncRNAs) regulate genes involved in inflammaging [167].

### DNA Methylation

DNA methylation is a prevalent epigenetic modification during aging, presented in the cytosine-phospho-guanine (CpG) dinucleotide promoter region [168]. Hypo-DNA methylation is due to a decrease in the activity of DNMTs, and the loss of DNA methylation triggers the activation of genes involved in inflammatory responses. At the molecular level, DNA methylation in undesirable genes is induced by oxidative stress which is itself aggravated by chronic inflammation, re-localizing DNMTs into guanine and cytosine-rich regions [169]. Additionally, tissue necrosis also contributes to DNA methylation in aging along with inadequate clearance of apoptotic cells impacting cell-free DNA. This unmethylated cell-free DNA is sensed as microbial DNA by DNA-sensing receptors, thus inducing inflammatory responses [170]. DNA methylation occurs with aging involving specific regions of the genome and this epigenetic changes can be linked with biological processes intricated in the aging [171].

Aberrant DNA methylation patterns associated with AD cause disruptions in DNA methylation at CpG sites, specifically in the genes viz*.* SORL1, ABCA7, HLA-DRB5, SLC24A4, and BIN1, suggesting that epigenetic regulation acts as a considerable part in the disease's progression and the overall aging of the brain [172]. The antioxidant potential of WS may be used in the treatment of several metabolic diseases which are linked with DNA damage, further preventing the cellular effects of inflammaging [161].

### Histone Modification

Histone proteins compactly pack the double strand DNA and form chromatin. The N-terminal of histones undergo epigenetic modifications like methylation, phosphorylation, acetylation, and ubiquitylation, mediating the activation or inhibition of the transcription of various genes involved in aging and age-associated diseases [171, 173].

The histone modifier polycomb protein group (PcG) is vital to the regulation of cellular senescence, and changes mediated by PcG lead to the repression of the participation of genes in cell cycle progression and the maintenance of cellular proliferation—potential targets for interventions in age-related diseases [174]. The interaction of activity-dependent neuroprotective protein (ADNP), sirtuin 1 (SIRT1) and their roles in regulating microtubules and histone methylation suggest that SIRT1 is associated with aging, whereas ADNP plays a key role in brain development and function. Further, mutation of ADNP leads to development of diseases like autism and AD. ADNP and SIRT1 interact with H3K79me2, HDAC2, YY1, BRG1, and WDR5 histones modifiers to affect histone methylation and deacetylation, thereby impacting chromatin structure and gene expression. Dysregulation of the interactions of ADNP and SIRT1 with histone modifiers can lead to aging and age-related diseases [175].

The effect of WS on the SIRT1 gene in the context of age-related circadian rhythm changes in old male Wistar rats was investigated. Results revealed that treatment with WS restores SIRT1 gene rhythms in the rats. As SIRT1 participates in the acetylation and deacetylation of histones through various histone modifiers, the restoration of SIRT1 activity is crucial for maintaining gene expression [176].

### Non-coding RNAs

ncRNAs are part of an emerging shift in epigenetics studies. They affect various biological transcriptional process through interaction with chromatin and modulate aging by acting as a mediator of epigenetic effects [168, 171]. MicroRNAs (miRNAs) alter the expression of inflammatory cytokines participating in inflammatory signaling pathways i.e., NF-κB/NLRP3 and IL-6, thus regulating inflammation [177]. Moreover, they modulate gene expression via interaction with epigenetic modifications, affecting age-related patterns [178]. The long non-coding RNA (lncRNA) FLJ46906 in aging fibroblasts is significantly involved in inflammatory responses, by regulating the transcription factors NF-κB and AP-1 which modulate the genes participating in the aging process [179].

Kim *et. al.* investigated the function of Mir-25 in the upregulation of COX-2 by treating rabbit articular chondrocytes with withaferin A. Withaferin A mediated Mir-25-induced inflammatory responses via inhibition of anti-Mir25 which further suppressed COX-2 expression [180]. In another study, withaferin A-mediated miRNA expression, specifically, miR-let-7c-5p upregulation, which inhibited breast cancer cell-derived mammospheres [181]. WS could be a potential therapeutic target in targeting specific signaling pathways in aging and aging-related diseases [182].

## Conclusions and Future Perspectives

The worldwide population is undergoing an aging drift and elderly people are more vulnerable to age related diseases, hence the enhanced mortality and morbidity. Chronic inflammation is closely related to escalating age, and it is important to comprehend the biological markers, epigenetic modifications, and signaling pathways involved in chronic inflammation and aging. Prevention and mitigation of aging-associated diseases and increasing quality-of-life has been the primary focus of aging research. In the past decade, increasing attention has been paid towards the use of nutraceuticals in combating age-related diseases. Moreover, the increasingly favorable attitude of the population towards nutraceutical use in combating age-related diseases cannot be ignored.

WS has been used in the traditional Indian medicine system since ancient times for its potential health benefits. WS possesses antioxidant potential against increasing oxidative stress with aging and has the ability to slow down the progression of inflammaging in older populations. Further, the anti-inflammatory and neurodegenerative properties of WS contribute to the modulation of age-linked diseases i.e., AD, PD and dementia which are most prevalent in older populations. Moreover, the potential effects of WS in the maintenance of the gut microbiome highlight a key role of WS in inflammaging.

This review summarizes the mechanisms of inflammaging, epigenetic modifications, aging and inflammation at the cellular level along with related diseases, while shedding light on WS’s regulatory signal transduction in the modulation of the AKT, NRF2 and NF-κB pathways of inflammaging. However, there is still limited evidence on the role of WS in inflammaging and more experimental evidence is required to support WS as a protagonist against inflammaging. Although WS extract shows positive effects against inflammation and aging, more research and clinical studies are needed, especially on enriched extracts of WS containing higher proportions of particular active components.

In conclusion, the jury is still out on the best way to reverse aging. Despite concerns about toxicity of certain WS extracts, this seems to be due to incorporation of leaf extracts in spurious sources as opposed to the beneficial root extracts of WS. The complexity of the regulatory cross-talk between molecular markers modulated by WS, and the different expression of some markers in distinct cell and tissue types add intrigue to the question of finding the best dose needed and the appropriate length of dosing with WS. Future clinical trials with a large cohort in a randomized trial with appropriate molecular phenotyping will likely better answer these questions.

## Data Availability

All the data generated in the current work has been included in the manuscript.

## Abbreviations

Akt: Serine/threonine kinase
AP-1: Activator protein 1
AMPK: Adenosine monophosphate-activated protein kinase
ARG1: Arginase-1
Apo-E: Apolipoprotein E
BAFF: B-cell activating factor
Bax: Bcl-2-associated protein x
Bcl-xL: B-cell lymphoma-extra large
Ca_v_1.2: Calcium channel, voltage-dependent, L type, alpha 1C subunit
cGAS-STING: Cyclic GMP-AMP synthase-Stimulator of interferon genes
CCAAT/ CEBPB: Enhancer-binding protein beta/ enhancer binding protein
CEBPB: Enhancer-binding protein beta
COL-II: Type II collagen
COX-2: Cyclooxygenase-2
CRP: C-reactive protein
DA: Dopamine
DOPAC: 3,4-Dihydroxyphenylacetic acid
ERK ½: Extracellular regulated kinases
GM-CSF: Granulocyte-macrophage colony-stimulating factor
HVA: Homovanillic acid
IDO1: Indoleamine 2,3-dioxygenase 1
iNOS: Inducible nitric oxide synthase
JNK: C-Jun N-terminal kinase
KEAP1: Kelch-like ECH-associated protein 1
LPS/TLR4: Lipopolysaccharide/Toll-like receptor 4
LRRK2: Leucine-rich repeat serine/threonine-protein kinase 2
MMP-13: Matrix metalloproteinase-13
mTOR: Mammalian target of rapamycin
MDSCs: Myeloid-derived suppressor cells
MAPK: Mitogen-activated protein kinase
NADPH: Nicotinamide adenine dinucleotide phosphate hydrogen
NF-κB: Nuclear factor kappa B
NLRP3: Nucleotide oligomerization domain-like receptor family pyrin domain-containing 3
Nrf2: Nuclear factor erythroid 2-related factor 2
PTEN: Phosphatase and tensin homolog deleted on chromosome ten
PI3K/AKT/FoxO3a: Phosphoinositide 3-kinase/protein kinase B/forkhead box class O 3a
PINK1: Phosphatase and tensin homologue-induced kinase 1
PMA: Phorbol 12-myristate 13-acetate
PSEN1: Presenilin 1
SOD: Superoxide dismutase
SERCA2: Sarcoplasmic/endoplasmic reticulum calcium ATPase 2
SARS-CoV-2: Severe acute respiratory syndrome coronavirus 2
SIRT3: NAD-dependent deacetylase sirtuin-3
SLC9A1: Solute carrier family 9 member A1
TNF-α: Tumor necrosis factor alpha
TDP-43: TAR DNA-binding protein 43
TNFSF12: Tumor necrosis factor ligand superfamily member 12
TGF-β: Transforming growth factor-β
TBNK cells: T and B natural killer cells
WA: Withaolide A
WAD: Withagenin A diglucoside
WS: *Withania somnifera*
ZBP1: Z-DNA-binding protein 1
